# The Effect of Chlorhexidine and Listerine® Mouthwashes on the Tensile Strength of Selected Absorbable Sutures: An* In Vitro* Study

**DOI:** 10.1155/2018/8531706

**Published:** 2018-11-13

**Authors:** Mohammed Alsarhan, Hourya Alnofaie, Rawan Ateeq, Ahmed Almahdy

**Affiliations:** ^1^Department of Periodontics and Community Dentistry, College of Dentistry, King Saud University, P.O. Box 60169, Riyadh 11545, Saudi Arabia; ^2^Dental Intern, College of Dentistry, King Saudi University, P.O. Box 60169, Riyadh 11545, Saudi Arabia; ^3^Department of Pediatric Dentistry and Orthodontics, College of Dentistry, King Saud University, P.O. Box 60169, Riyadh 11545, Saudi Arabia

## Abstract

**Background:**

Suturing plays a critical role in the healing of surgical wounds. The tensile strength of suture materials indicates the ability of the material to withstand stress during knotting and protect the wound during an extended period of healing.

**Objective:**

An in vitro study was conducted to determine the effect of two commercially available mouthwashes on the tensile strength and breakage mode of two absorbable intraoral sutures.

**Materials and Methods:**

Two common absorbable sutures, Vicryl® and Monocryl®, both with 4-0 and 5-0 gauges were used. A total of 400 specimens were sutured around rubber rods and immersed in three thermostatically controlled experimental conditions: artificial saliva, 0.2% chlorhexidine gluconate (Parodontax® extra), and essential oils-based rinse (Listerine® Zero™), and these were compared to a nonimmersed dry condition. All specimens were stored in an incubator at 37°C. Tensile strengths were assessed after days 1, 3, 7, 10, and 14 of immersion using a universal Instron® testing machine. The maximum load for suture breakage and the location of the point of breakage were assessed.

**Results:**

Unlike Monocryl® 4-0, the tensile strength of both gauges of Vicryl® sutures significantly increased in chlorhexidine and Listerine®. There was a significant decrease in the strength for all suture types after day 10, regardless of the immersion solution. Listerine® significantly reduced the tensile strength of Monocryl® 5-0.

**Conclusion:**

Oral surgeons and periodontists should be cautious when prescribing commercial mouthwashes for patients relative to their selection of suture materials. However, further studies are needed to understand the molecular changes in sutures when exposed to chemical solutions found in mouthwashes.

## 1. Introduction

Suturing plays a critical role in maintaining the tissue integrity of surgical wounds. A key aspect of acceptable wound closure is to assure that sustained approximation of flap margins remains stable over a certain period. This allows for a favorable level of tissue healing and fulfills a positive treatment outcome [1]. Failure in achieving wound closure may lead to delayed healing or wound dehiscence with subsequent functional and aesthetic complications [2].

The understanding of suturing materials and techniques is essential to the practice of surgical dental procedures. Several types of suture materials in the oral cavity have been evaluated in the literature [3–6]. These materials are under continuous mechanical forces from mastication, speech, facial expressions, and alteration in pH levels, bacterial proteolytic enzymes, saliva, and vascularization [7–9]. While it is difficult to reduce mechanical forces across suture lines in the oral cavity, the most important characteristic of an acceptable suture material is the ability to protect wounds for optimal healing with minimal or no tension [3, 10, 11]. In addition, many surgeons have attempted to limit tension by advising a liquid or soft diet or by minimizing chewing and speech during postoperative healing [12].

Sutures are divided mainly into categories of synthetic or natural, and absorbable or nonabsorbable, based on their degenerative and resorptive capacity. Poliglecaprone 25 (Monocryl®) is a monofilament synthetic absorbable surgical suture prepared from a copolymer glycolide and epsilon-capro-lactone while polyglactin 910 (Vicryl®)) is a multifilament absorbable synthetic coated suture composed of a copolymer made from 90% glycolide and 10% L-lactide.

Monocryl® and Vicryl® sutures have been widely used due to their various physical and biomechanical features including their degradation rate, reduction of adherent bacteria biofilm, and better healing response [13–15]. These unique features contribute to their popularity and usage in various oral and periodontal surgical procedures.

The tensile strength of suture materials is one of the important mechanical characteristics that indicate its ability to withstand stress during knotting [16]. Furthermore, maintaining the basal tensile strength of the suture material is of absolute importance for stabilizing and securing the sutured flaps at the time of surgery until the time of removal. Clinically, inflammation of the surgical flaps during wound healing may exert a certain degree of tension on wound edges and cause subsequent bridging of flap margins [1]. Thus, it is of utmost importance to maintain the approximate wound margins via sutures that have an acceptable level of tensile strength with minimal tissue reaction. Sutures with insufficient tensile strength during the healing phase could break, causing poor adaptation of the margins, hematoma, and subsequent deterioration of the affected site [17, 18]. It is suggested that suture selection should be primarily based on the physical and biomechanical properties that will promote better local wound healing [19, 20].

Several studies report that a suture's tensile strength may be affected by specific solutions or consumed fluids. An experimental study by* Ferguson *et al. shows a progressive loss of tensile strength in Vicryl® suture materials when subjected to saliva, bovine milk, and soy milk over a period of 35 days [12]. Saliva-soaked specimens show a more rapid loss of tensile strength than the other soaking liquids [12]. Another study reports that Vicryl® shows better breaking strength compared to natural sutures. This is especially evident after immersion in physiological and acidic pH solutions [11]. Moreover, a recent report suggests that antiseptic solutions have an impact on the failure load of sutures used in knee surgery [21].

However, to the best of our knowledge, no study has compared the strengths of Monocryl® and Vicryl® suture materials over time when exposed to commercial oral mouthwash solutions.

Therefore, the current study aims to evaluate the tensile strength and breaking point of Monocryl® and Vicryl® sutures in association with two different commercial types of mouthwashes (Parodontax® and Listerine® Zero™), an orally simulated environment (artificial saliva) and a nonimmersed dry condition during a two-week period.

## 2. Materials and Methods

### 2.1. Suture Specimens

Two types of absorbable sutures, multifilament coated polyglactin 910 (VICRYL®* PLUS - *Ethicon™) and monofilament poliglecaprone 25 (MONOCRYL®* PLUS - *Ethicon™), were used in this study. For each type of suture, two gauges (4-0 and 5-0) were selected. At least 20 suture packs of each suture material and gauge (Vicryl® 4-0, Vicryl® 5-0, Monocryl® 4-0, and Monocryl® 5-0) were employed using a stratified randomized selection process. Each suture pack was utilized to create five suture specimens tied around a single custom-made rubber rod with a diameter of 4 mm. These rubber rods were fabricated by molding and curing a vinyl-polysiloxane material (Deguform® Plus). Four rubber rods, each containing 5 suture specimens of each material and gauge, were placed in a plastic container labeled with the experimental condition name and sample number.

**Figure 1:**
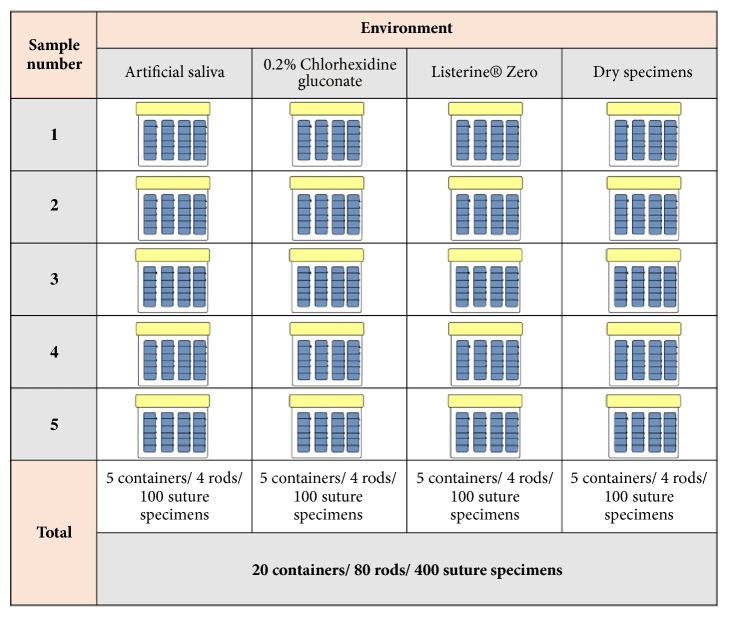
Overview of sutures and media distribution.

This resulted in a total of 400 suture specimens (Figure 1). Each tested suture specimen was tied using knots consisting of an initial triple-wrap throw (surgeon's throw) followed by two square throws, yielding a 3:1:1 pattern. Based on 45 and 70 cm suture lengths, each sample was used as follows: (a) 40 mm as positive control (artificial saliva), (b) 40 mm for each test group (0.2% chlorhexidine and Listerine® mouthwashes), and (c) 40 mm as negative control (dry nonimmersed specimens). The samples were contained in 500 ml sterile plastic containers and remained in tension. All specimens that slipped or lost tension during pretesting were recorded.

### 2.2. Experimental Conditions

Three thermostatically controlled experimental conditions were used for suture immersion. The tested media included artificial saliva as a positive control group (CG-1), 0.2% chlorhexidine gluconate (Parodontax® extra) (TG-1), and an essential oils-based rinse (Listerine® Zero™) (TG- 2). A dry condition (nonimmersed specimens) was used as a negative control group (CG-2). All solutions were prepared and maintained at standard pH levels throughout the timeframe as follows: 8.6 for artificial saliva, 8.4 for chlorhexidine, and 4.5 for Listerine® as measured by an HQ411d HACH® Laboratory pH Meter. All media were stored in an incubator at 37°C. During the study period, rubber rods were removed from their containers, rinsed with distilled water, and then replenished with media every 48 hours.

### 2.3. Artificial Saliva Preparation

Artificial saliva was prepared by mixing 100 mL each of 25 mM K_2_HPO_4_, 24 mM Na_2_HPO_4_, 1.570 mM KHCO_3_, 100 mM NaCl, and 1.5 mM MgCl_2_, followed by adding 6 mL of 25 mM citric acid and 100 mL of 15 mM CaCl_2_. The solution was kept in a dark container until the execution of the experiment.

### 2.4. Tensile Strength

Tensile strengths of the suture samples were tested at specified time periods: 1, 3, 7, 10, and 14 days after immersion. During the testing day, one specimen was gently removed from the rubber rod using utility pick-up pliers (Hu-Friedy Mfg. Co., LLC Chicago, IL, USA) for testing, whereas the rest of specimens were left untouched, to be tested on subsequent days. A total of 80 suture specimens were evaluated on each testing day.

**Figure 2:**
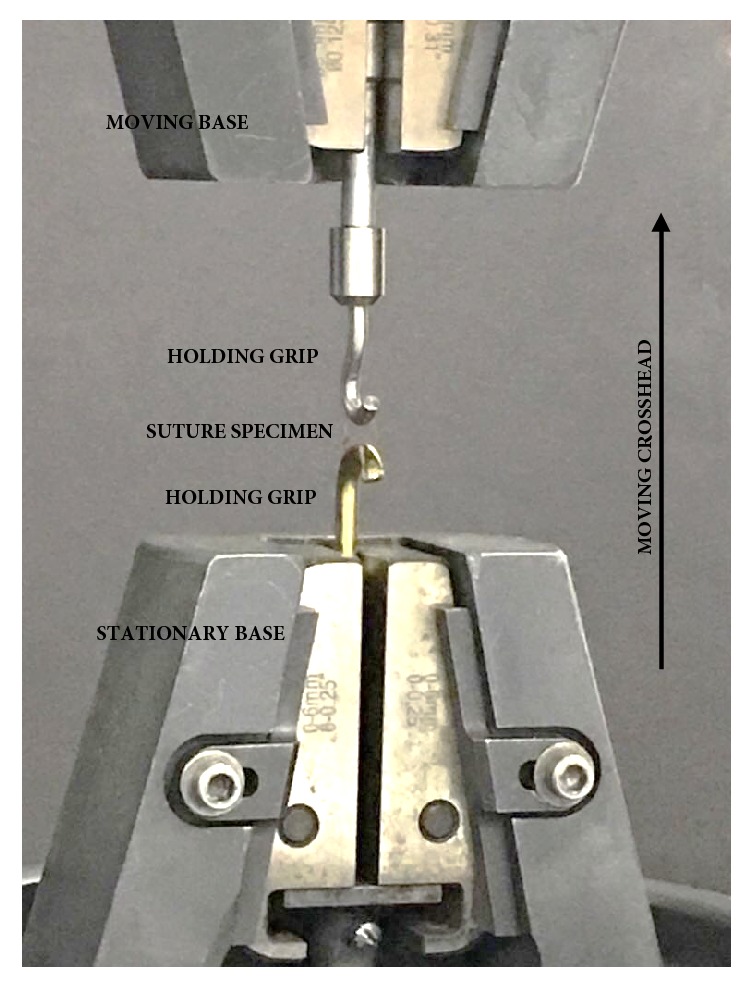
Photograph displaying how suture specimen is mounted on Instron® machine prior to tensile strength testing.

Tensile strength of the suture samples was measured using a universal Instron® Testing System (model 5965 with 5 kN (1125 lbf) capacity) at a cross-head speed of 10 mm/minute. During the delivery of the suture specimen for testing, the knot was located midway between the base and the hook of the Instron® machine (Figure 2). Additionally, each specimen was stretched to failure and the tensile strength was recorded in (MPa) and tabulated for analysis (Table 1). Moreover, the point of breakage was located and documented.

### 2.5. Statistical Analysis

The results of continuous measurements were presented as the mean and standard deviation (min-max), with significance assessed at *α* = 0.05, estimated standard deviation of 1, and power of 0.83. The sample size for each group under investigation was 20. A two-factor analysis of variance (ANOVA) followed by Tukey's post hoc analysis was conducted to assess the tensile strength of materials over time and media. A Pearson Chi-square test was used to observe the association between categories of point breakage. Independent variables were suture material and gauge, immersion time, media type, and temperature. Data management and analysis were performed using SPSS 23.0.

## 3. Results

### 3.1. Tensile Strength

**Figure 3:**
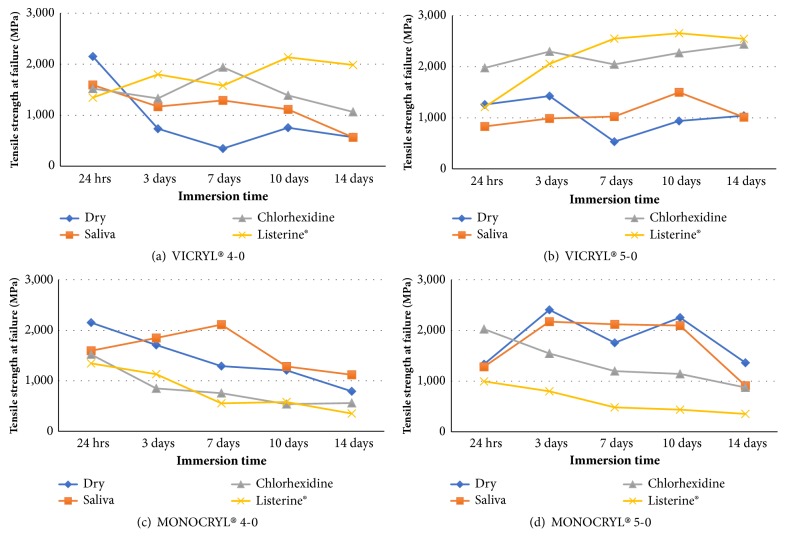
Tensile strength of dry sutures compared to immersion in saliva, chlorhexidine, and Listerine solutions for up to 14 days for (a) Vicryl 4-0, (b) Vicryl 5-0, (c) Monocryl 4-0, and (d) Monocryl 5-0.

The mean tensile strength of absorbable sutures was statistically different in the overall model for the tested experimental conditions and times (p<0.01). Compared to its strength after 24 hours of immersion, the tensile strength of Vicryl® 4-0 (Figure 3(a)) after ageing was significantly different for samples immersed in each solution (and dry) at different times (p= 0.006), and all strengths decreased significantly (0.033) on day 14, except for Listerine®. The changes were not significant across the other tested times. There was no significant difference in the tensile strength of Vicryl® 4-0 sutures immersed in saliva (CG-1) and those of the dry, nonimmersed condition (CG-2) (p=0.563). However, chlorhexidine (TG-1) and Listerine® (TG-2) resulted in a significant increase in the tensile strength when compared to the dry condition (CG-2) (p=0.022 and p<0.001, respectively). There was no significant difference between chlorhexidine (TG-1) and Listerine® (TG-2) groups (p=0.315) for Vicryl® 4-0 suture tensile strengths except on day 14.

For Vicryl® 5-0 (Figure 3(b)), no significant difference was found in the tensile strength when samples were immersed in different solutions at different times (p=0.277). However, chlorhexidine and Listerine® mouthwashes resulted in a significant increase in the tensile strength when compared to the artificial saliva medium (CG-1) and the dry nonimmersed condition (CG-2) (p<0.01) when the time factor is excluded.

For Monocryl® 4-0 sutures (Figure 3(c)), tensile strength decreased after immersion in chlorhexidine media (TG-1) and Listerine® media (TG-2) compared to the tensile strength after immersion in an artificial saliva medium (CG-1) and the dry nonimmersed condition (CG-2). However, this reduction was not significant (p=0.641) for the tested times. Regardless of the immersion media, a significant reduction of strength was observed starting from day 10 (p=0.004). Similarly, Monocryl® 5-0 sutures (Figure 3(d)) had a significant reduction in the tensile strength when immersed in Listerine® media (p<0.01). No significant differences were noted for the other tested solutions.

### 3.2. Point of Breakage

**Figure 4:**
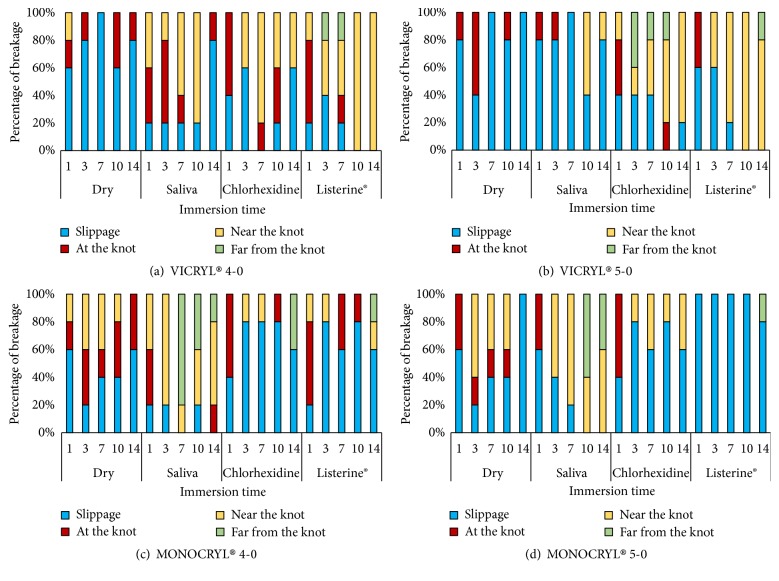
The point of breakage for dry sutures compared to sutures immersed in saliva, chlorhexidine, and Listerine for up to 14 days of immersion using (a) Vicryl 4-0, (b) Vicryl 5-0, (c) Monocryl 4-0, and (d) Monocryl 5-0.

For Vicryl® 4-0, the dry condition (CG-2) showed significantly more slippage of the suture when compared to the other media (p<0.001) (Figure 4(a)). For the suture immersed in Listerine® (TG- 2), the breakage was not typically associated with the knot after 7 days when compared to other conditions (p=0.041). No statistically significant difference was found between saliva (CG-1) and chlorhexidine (TG-1) (p=0.154).

Similarly, Vicryl® 5-0 in a dry condition or in saliva (CG-1) showed significantly more slippage when compared to the other two conditions (p=0.027 and p=0.049, respectively) (Figure 4(b)). No significant differences were found between saliva (CG-1) and the dry condition (CG-2) (p=0.054) and between chlorhexidine (TG-1) and Listerine® (TG-2) (p=0.608). The latter two solutions showed more breakage along the length of the suture (both near the knot and farther from the knot), as opposed to right at the knot (p=0.021).

On the other hand, Monocryl® 4-0 sutures immersed in chlorhexidine (TG-1) and Listerine® (TG-2) showed significantly more slippage of the suture when compared to the other conditions (p=0.050 and p=0.010, respectively) (Figure 4(c)). The sutures immersed in saliva (CG-1) showed more breakage along the length of the suture (both near the knot and farther from the knot), as opposed to right at the knot when compared to the other three media (p=0.010). Similar results were found for Monocryl® 5-0 (Figure 4(d)). Dry (CG-2) and saliva (CG-1) media resulted in significantly more breakage along the length of the suture and not at the knot (p=0.033 and p=0.021, respectively).

## 4. Discussion

The present study aimed to test the effect of chlorhexidine and Listerine® mouthwashes on the tensile strength of polyglactin 910 (Vicryl®* PLUS*) and poliglecaprone 25 (Monocryl®* PLUS*) sutures, for both 4-0 and 5-0 gauges. The selection of suture materials was based on their versatility and popularity for various oral and periodontal surgical procedures. In addition, the selection of mouthwashes was based on the frequent prescription of chemotherapeutic agents to control plaque formation [22]. We chose to study artificial saliva because previous studies observed a possible effect on suture strengths, while a dry condition was used to evaluate the unsoaked tensile strength of the same sutures over time. The duration of this study and the testing times were based on the clinical relevance of common oral surgical procedures.

Monocryl®* PLUS *and Vicryl®* PLUS *sutures were absorbed by the process of hydrolysis. At the 14-day postimplantation period, Vicryl® sutures retained more than two-thirds of their original tensile strength, while Monocryl® sutures retained around one-third of the original strength [23]. A strong relationship between suture degradation and tensile strength has been reported in the literature under controlled in vitro and in vivo settings [3, 6, 7]. The current study found that the mean tensile strength was significantly different as a function of immersion media and time frame (Section 3.1).

The specific methodology followed in this investigation was designed to assure consistency in the types of materials tested under different conditions and time frames. The acidity or alkalinity of solutions in contact with the sutures plays a significant role in the resorption of suture materials. Throughout the time frame of the study, pH levels of all solutions were consistently measured and maintained. The knot configuration of sutures is another important factor that influences suture stability [24]. The present study used the surgeon's knot in all samples to reduce knot untying and ensure stability.

Among the 4-0 sutures, Vicryl® strength decreased significantly (0.033) on day 14 except in Listerine®. Furthermore, there was a greater tendency for suture breakage, rather than untying, in the Listerine® group. On the other hand, Monocryl® showed an insignificant strength reduction as a function of media over time. However, slippage of sutures significantly increased when Monocryl® was immersed in chlorhexidine and Listerine® solutions. This phenomenon might be explained by the ability of multifilament braided sutures such as Vicryl® to resist knot untying compared to monofilament sutures like Monocryl® under increased tensile loads [25]. Despite the methodological differences,* Kim *et al. reported that monofilament sutures showed a tendency to untie easily after being loaded to failure [25]. In addition, the effect of creating a knot reduces the mechanical properties of sutures [24, 26].

The findings of our current experiments are consistent with those studies for all sutures types. Moreover, the tensile strength of synthetic multifilament absorbable sutures is sustained under acidic or neutral pH levels [27]. Due to their morphology, Monocryl® sutures tend to lose tensile strength in a shorter time [27]. In an evaluation of the tensile strength of sutures under various pH conditions, different absorbable and nonabsorbable sutures were immersed in saline buffer solutions with pH ranging from 3.0 to 10.0 [11]. The study found that synthetic multifilament absorbable sutures in alkaline environments experience a degradation of the tensile strength and resorption rate, while more acidic or neutral pH conditions tend to retain their breakage strength. Vicryl® sutures in our investigation displayed similar results when immersed in Listerine® mouthwash. However, Monocryl® sutures showed a notable loss of strength when immersed in both chlorhexidine and Listerine® mouthwashes. Because the suture size was similar for both Vicryl® and Monocryl®, this inconsistency in degradation may be due to the differences in their physical structures where Vicryl® is a multifilament braided suture, giving it more resistance to hydrolytic degradation over longer time periods.

It is a common practice for oral surgeons to prescribe antiseptic mouthwashes following surgical procedures, but the effect of various antiseptic mouthwashes on sutures has not been entirely tested. Contradicting the current study hypothesis which stated that antiseptic commercial mouthwashes had an effect on the tensile strength of Vicryl® and Monocryl® suture materials, previous clinical studies found no significant difference in the loss of strength of Vicryl® and Vicryl Rapide® sutures when subjected to chlorhexidine mouthwash [28, 29]. This discrepancy may be attributed to the limited duration of exposure of the sutures to chlorhexidine mouthwash in the previously cited clinical studies. In addition, longevity rather than tensile strength was measured in those studies.

The findings of the current study revealed a significant difference between the two gauges of Vicryl®, and between Vicryl® 5-0 and Monocryl® 4-0. The gauge 5-0 strength of both types of sutures was significantly higher than for gauge 4-0. This result is different from previous research that evaluated both gauges of Vicryl® sutures, in which the tensile strength of the 4-0 sutures was statistically insignificant but higher when immersed in a simulated oral environment [3]. In another study, a significant difference in tensile strength between the two gauges was observed, favoring 4-0 Vicryl® sutures at day 10, but a negligible difference was noted at day 14 [30].

Although there was no significant difference in the strength of Vicryl® gauges between chlorhexidine and Listerine® in our study, Vicryl® 4-0 in Listerine® exhibited more widespread suture breakage than slippage, suggesting more resistance and stability of the knot. In contrast, Monocryl® 5-0 was significantly weaker when immersed in Listerine®, and slippage was recorded more frequently than breakage. The breakage point correlated to the tensile strength values for the tested solutions, possibly due to suture morphology where monofilament sutures degrade faster, leading to increased knot slippage [27].

The current study has demonstrated a significant difference in the tensile strength of suture materials depending on their environmental conditions. Interestingly, Listerine® and chlorhexidine mouthwashes substantially affected the physical properties of the tested suture materials. However, one of the limitations of this study was the inability to evaluate other important factors in the oral environment where saliva interacts with serum fluids within gingival flaps. Furthermore, this being an in vitro experiment, results may not be completely applicable to an in vivo environment. Factors such as dietary habits, smoking, comorbidities, and medications can potentially alter the oral pH level and cause changes at the molecular level of sutures, and hence may influence the study results.

## 5. Conclusions

In conclusion, our findings suggest more evaluation of how commercial mouthwashes may influence the physical characteristics of the suture strength and stability and its impact during the healing period of surgical wounds. The current study suggests that Listerine® mouthwash can be prescribed safely after using either Vicryl® 4-0 or 5-0 sutures or Monocryl® 4-0 sutures.

However, Monocryl® 5-0 sutures performed better when 0.2% chlorhexidine gluconate mouthwash was used. We recommend further testing with in vivo experiments in order to understand the molecular changes of sutures when exposed to chemicals in mouthwashes and to confirm the methods and clinical outcomes.

## Figures and Tables

**Table 1:** Summary of the mean values + standard deviation (SD) of sutures tensile strength in Mega Pascal (MPa) in relation to different media and time points.

SUTURE	MEDIA	TIME POINTS				
		24 hours	3 days	7 days	10 days	14 days
VICRYL 4-0	DRY	2151 (1257)	735 (148)	346 (311)	755 (462)	571 (708)
	SALIVA	1594 (805)	1168 (586)	1291 (454)	1115 (558)	565 (377)
	CHX	1522 (1219)	1331 (681)	1940 (174)	1388 (470)	1064 (717)
	LISTERINE	1345 (583)	1798 (739)	1580 (888)	2134 (154)	1984 (138)

VICRYL 5-0	DRY	1260 (1435)	1425 (395)	533 (134)	938 (579)	1040 (589)
	SALIVA	831 (448)	988 (555)	1025 (580)	1499 (666)	1011 (652)
	CHX	1978 (1019)	2298 (1048)	2045 (1031)	2271 (787)	2439 (177)
	LISTERINE	1211 (893)	2057 (995)	2550 (785)	2656 (135)	2546 (263)

MONOCRYL 4-0	DRY	2151 (1257)	1709 (386)	1291 (518)	1208 (777)	793 (239)
	SALIVA	1594 (805)	1847 (831)	2113 (149)	1285 (574)	1120 (170)
	CHX	1522 (1219)	850 (803)	756 (562)	538 (312)	560 (270)
	LISTERINE	1345 (583)	1131 (741)	554 (303)	580 (527)	352 (261)

MONOCRYL 5-0	DRY	1334 (943)	2407 (1007)	1757 (879)	2255 (567)	1363 (793)
	SALIVA	1284 (1035)	2175 (1453)	2121 (682)	2097 (117)	910 (192)
	CHX	2026 (1223)	1546 (880)	1197 (904)	1143 (507)	874 (548)
	LISTERINE	995 (406)	800 (310)	481 (226)	437 (179)	353 (178)

## Data Availability

The data used to support the findings of this study are available from the corresponding author upon request.
